# A study on the effects of perceived playfulness and emotional support in interactive learning environments for German language acquisition—A role-playing library system case

**DOI:** 10.3389/fpsyg.2022.1073985

**Published:** 2022-12-28

**Authors:** Yunshuo Yang, Jiacheng Lin, Tong Chen, Shuyuan Lin, Jiangjie Chen, Wei Miao, Wei Wei, Hanchu Sun, Jie Sun, Chao Gu

**Affiliations:** ^1^College of Foreign Languages and Cultures, Xiamen University, Xiamen, Fujian, China; ^2^Department of Computer Science and Information Engineering, National Taiwan University, Taipei, Taiwan; ^3^Michael Smurfit Graduate Business School, College of Business, University College Dublin, Dublin, Ireland; ^4^Department of Media Design, Tatung University, Taipei, Taiwan; ^5^School of Design, Jiangnan University, Wuxi, Jiangsu, China; ^6^School of Textile Garment and Design, Changshu Institute of Technology, Changshu, China; ^7^Faculty of Industrial Design Engineering, Delft University of Technology, Delft, Netherlands; ^8^College of Arts and Design, Zhejiang A&F University, Hangzhou, China; ^9^Department of Culture and Arts Management, Honam University, Gwangju, South Korea

**Keywords:** emotional support, perceived playfulness, flow experience, learning interest, continuous learning intention

## Abstract

**Introduction:**

This study examines the emotional support offered by the non-player characters (NPCs) in an interactive learning environment, as well as the effects of the perceived playfulness of the interactive system on German language learning.

**Method:**

We developed a role-playing library system to serve this purpose. 2,377 Chinese Internet users were surveyed using online questionnaire.

**Results:**

A theoretical model of emotion- driven learning (ELM) was proposed based on the analysis results of valid recovered data. Additionally, NPCs were found to be effective in improving learning outcomes through emotional support.

**Discussion:**

An interactive education system may be able to enhance the perceived playfulness of learning in order to enhance the learning experience.

## Introduction

### Research background

Since 2015, there have been progressively more people learning German around the world, especially in China. The Chinese Ministry of Education declared in 2018 that German would be a topic in college entrance exams, which in part sparked Chinese students’ interest in studying German. Peer learning is regarded as being crucial to learning a foreign language (Ma, 2020). Due to the time, space, and social requirements of peers to attain high levels of coordination, this strategy, however, presents numerous problems. It should be emphasized that there is disagreement about whether simultaneous peer learning is effective for online German instruction. For instance, a research testing augmented reality picture books for learning German revealed no discernible difference between peer and independent learners’ opinions of the augmented reality picture books (Gu et al., 2022b). The development of user behavior research and educational technologies may depends greatly on incorporating the non-player character (NPC) into the interactive learning system and evaluating the real learning effects.

The importance of the digital transformation of education is rising in the modern world (Sillat et al., 2021). The education sector has started to focus a lot of attention and study on encouraging the digital evolution of education, especially in the context of COVID-19. Researchers discovered that employing digital technology in an art history course, strategies including spherical video-based virtual reality helped students attain more favorable learning outcomes, self-regulation, meta-cognitive awareness, and self-efficacy (Wu et al., 2021). As well, it was found that augmented reality technology can have a greater positive impact on learning outcomes after comparing the learning level of engineering students’ laboratory skills and the cognitive load in the laboratory (Singh et al., 2019). According to these findings, interactive technology creates new opportunities for the creation of online courses. Without face-to-face interaction, digital technology offers more convenient educational opportunities and encourages greater conversation on the importance of having fun while teaching and learning. Research shows that emotional support may even have a greater positive impact on academic performance than financial support (Borup et al., 2020). Previous studies on education often focus on the influence of emotional support from teachers, classmates, family and society on students’ learning. According to research on college students’ experiences with the remote teaching system, while students can sense the teachers’ empathy and concern during the online learning process, they do not receive the same level of emotional support from their peers, which lowers their motivation and concentration (Shin and Hickey, 2021). This suggests that in order to learn effectively in interactive learning environments, students may require a variety of emotional supports. On the other hand, interesting teaching activities are believed to enhance the communication between teachers and students (Padilla-Meléndez et al., 2013). Students’ willingness to participate in digital education was enhanced in the interesting learning process. But the interesting classes may have diverted students’ attention and failed to improve their attitude towards learning (Wang et al., 2021).

Researchers and designers have researched the evolution of digital education and interactive learning environments in great detail, but nobody has yet assessed the emotional support and deliberate fun that NPCs provide to the study of foreign languages, particularly German. Users that have negative views perform worse in robot-assisted language learning because they are less motivated to interact and speak with the robot, according to research (Kanero et al., 2022). It illustrates the necessity of studying the user behavior of a teaching system. Designing learning systems with user needs in mind helps to encourage positive views and lessen negative feelings. This study developed a role-playing library system to evaluate the effectiveness and efficiency of users’ German learning, as well as the contribution of emotional support from NPCs and the system’s playfulness design. Users of our digital library can interact with non-player characters (NPCs) that offer emotional support for learning and psychological welfare while reading German textbooks in a role-playing mode.

In general, our research focuses on two urgent problems that are connected to interactive learning environments. Or, to put it another way, whether the emotional support offered by NPC can enhance the user’s learning status and whether a perceived increase in playfulness will benefit the online curriculum. Making decisions based on actual experience is frequently tough for interaction designers and foreign language teachers. In fact, providing emotional support is frequently considered as listening and empathizing with another person (Klyver et al., 2018). Additionally, some teachers’ severe and serious teaching methods clash poorly with the lively and playful classroom environment (Poai, 2020). This study quantitatively analyzes user behavior and offers design suggestions. The designer can successfully select the function and style that should be stressed in a foreign language education system based on the study’s findings. To support teachers of foreign languages in raising the caliber of their instruction and the efficiency of classroom learning, this study have created a strong hypotheses framework for the theoretical development of interactive educational environments by examining the interactions between the various factors.

### Research purpose

In this study, a role-playing library system is developed to assist users in learning German. The purposes of the research include the following two aspects. Firstly, the developed system is used to assess if the emotional support offered by NPCs can successfully increase users’ learning interest and continuous learning intention of learning German in an interactive learning environment. Secondly, whether the perceived playfulness affects the user’s learning interest and continuous learning intention is verified.

## Theoretical framework and research hypotheses

**Figure 1:**
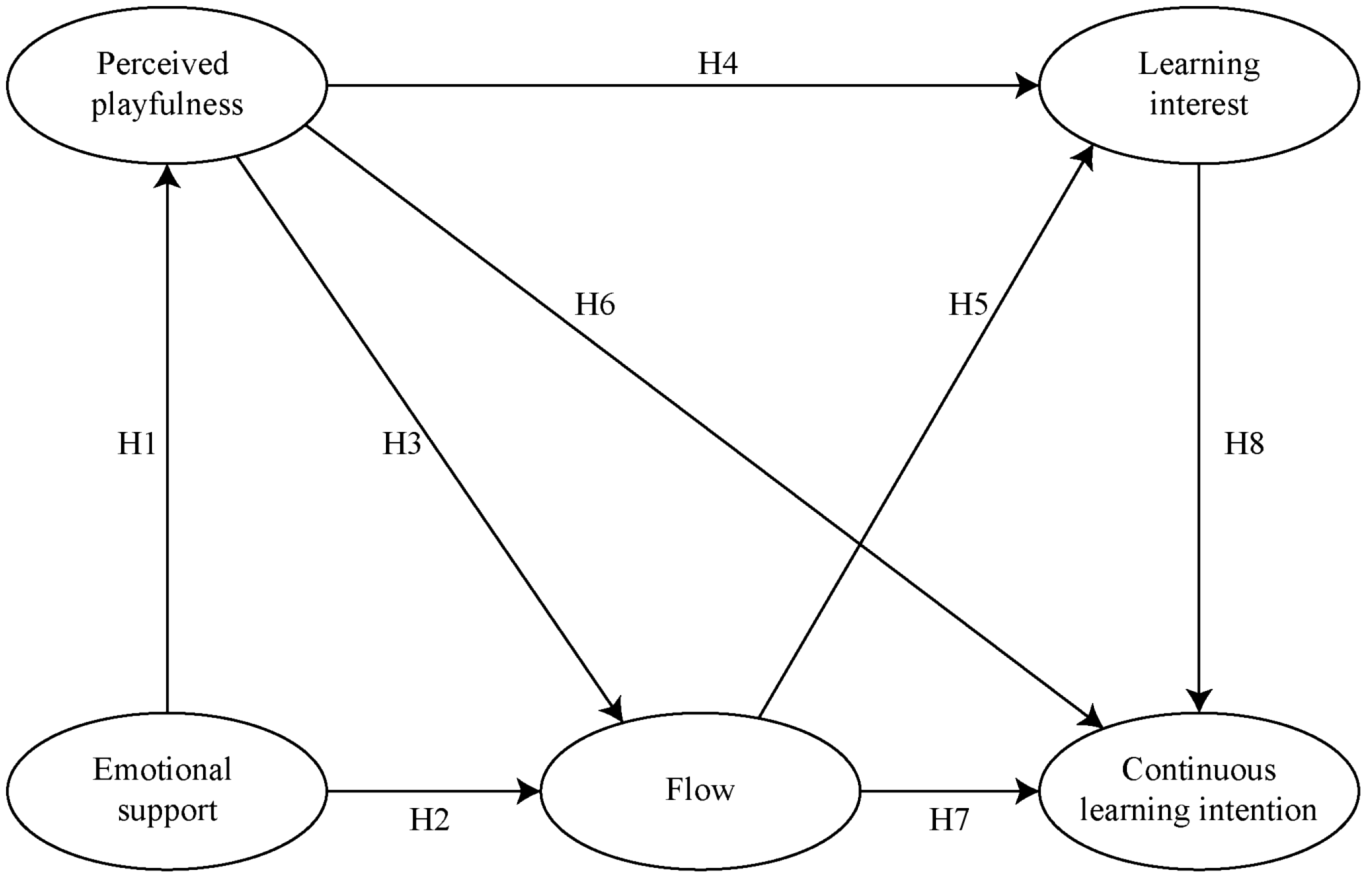
Hypotheses of the study.

This study examined users’ perceptions and behaviors in an interactive German language learning environment. Five dimensions, emotional support, perceived playfulness, flow, learning interest, and continuous learning intention, were included. This study proposed a relationship framework about the path relationship between dimensions, and all the hypotheses were tested. Figure 1 illustrates the model of hypotheses.

### Emotional support

The concept of emotional support refers to the process of gaining empathy from others and feeling comfortable with them (Green et al., 2021). Providing emotional support involves expressing care and concern for another individual, particularly when he or she is in a negative mood (Heilat and Seifert, 2019). Someone who offers emotional support exhibits empathy and concern for the recipient. Researchers have demonstrated that educators’ positive emotional support behaviors positively affect students’ learning outcomes (O’Hare et al., 2020). Students and teachers benefit from emotional support during learning and teaching activities. This type of assistance from a person necessitates some social skills, hence the student must also be able to assist others. Young people often experience social anxiety during interpersonal interactions (Meng et al., 2021). As a result, they turn to digital technology for emotional support (Prescott et al., 2019). The psychological needs of obtaining emotional support and avoiding human engagement throughout the learning process are both addressed by interactive learning environments. In this approach, students engage with NPCs through role-playing games. The effectiveness of this emotional support provided by NPCs as a teaching aid, however, has yet to be proven.

### Perceived playfulness

According to this definition, perceived playfulness is a combination of the user’s concentration, their curiosity during interaction, as well as their feelings of pleasure and enjoyment (Balkaya and Akkucuk, 2021). According to previous studies, perceived playfulness can enhance users’ willingness to use augmented reality technology in human-computer interaction. For instance, there are studies have examined the use of augmented reality in shoe shopping and digital museum exhibits that utilizing augmented reality (Jiang et al., 2022a,b). In the field of interactive education, perceived playfulness also plays an important role. Perceived playfulness is one of the most important factors that determines how users behave when learning with mobile technology (Nikolopoulou, 2018). However, it is important to note that interesting content may undermine the seriousness of classroom instruction. Therefore, when designing the digital education system, designers should consider elements of reasonable combination of interests. It is noteworthy that in kinship relationships, strong and robust emotional support contributes to the playfulness of interaction (Zhang et al., 2019). It is necessary to explore whether the feelings triggered by such interaction can enhance user’s experience within the digital education system. Therefore, the following hypothesis is proposed:

*Hypothesis 1 (H1)*: Emotional support from NPCs in an interactive learning environment can have a significant positive influence on perceived playfulness.

### Flow

An experience of flow is defined as the state of mind in which the user judges the difficulty of the task and his own ability to achieve it (Guerra-Tamez and Franco-García, 2022). In the flow state, the user loses track of time and changes in the environment because he is so focused on the task. The flow experience is different from immersion in that it is a state of all or nothing, whereas immersion is a sense of distinct levels (Cairns et al., 2014). Flow is a state of extreme positivity (Frochot et al., 2017). It is only possible for users to be entirely immersed in the flow experience, or not at all. As a result, interaction designers must completely take into account both user needs and potential flow state triggers. An examination of athletic competition and training found that athletes’ flow experiences were influenced by emotional antecedents during the season (Cece et al., 2022). Thus, changes in mood may be related to flow status. Additionally, previous research suggests that the flow experience is related to the user’s perception of playfulness (Triberti et al., 2021). If the user considers the participation process fascinating, the chance of flow triggering may increase. This study examined the influence of emotional support and perceived playfulness on users’ flow experience in interactive German learning as a means of verifying the possible influence relationship between these two groups of variables. Therefore, the following hypotheses are proposed:

*Hypothesis 2 (H2)*: Emotional support by NPCs in an interactive learning environment can have a significant positive influence on flow experience.

*Hypothesis 3 (H3)*: Perceived playfulness in an interactive learning environment can have a significant positive influence on flow experience.

### Learning interest

The term interest refers to the physiological response that occurs when one is confronted with a variety of objects, such as people, events, environments, etc. Interest shows up through behavioral modifications in attention, effort, and emotion (Tan et al., 2021). Generally speaking, a student’s interest in learning can be described as their willingness and desire to participate in class activities as well as their attitude while learning (Lange, 2018). When students are interested in learning, they are more likely to focus and study hard in a positive manner. Designing instructional materials and instructional strategies is done to increase students’ interest in learning during teaching activities (Gu et al., 2022c). Previous studies have demonstrated that interactive teaching methods are among the most crucial elements that support students’ learning environments (Andreani and Ying, 2019). Interactive technology has allowed educators to use interactive learning to stimulate students’ interest. A few studies have demonstrated positive effects during interaction. For example, using interactive teaching tools, preschoolers can improve their literacy and numeracy through playful learning (Rogowsky et al., 2018). Facial recognition can be used in teaching cases to trigger flow status and improve learning interest (Gu et al., 2022a). In any case, it is critical to remember that a successful and enjoyable experience can sometimes lead to students concentrating only on the interaction itself and ignoring the significance of the learning content (Triberti et al., 2021). It is necessary to examine the effects of perceived playfulness and flow on students in order to increase their learning interest. Therefore, the following hypotheses are proposed:

*Hypothesis 4 (H4)*: Perceived playfulness in an interactive learning environment can have a significant positive influence on learning interest.

*Hypothesis 5 (H5)*: Flow experience in an interactive learning environment can have a significant positive influence on learning interest.

### Continuous learning intention

The concept of continuous intention refers to the intent of the user to continue acting in the same manner as before (Dehghani, 2018). The concept of continuous learning intention in educational research refers to the intention of students to continue their learning state after a period of learning and to gain new knowledge and experience after the period of learning has ended. It is a measure of the likelihood that an individual will continue to learn on a subjective basis (Al-Samarraie et al., 2018). A longer period of time and regular learning result in systematic and in-depth learning. Previous studies have attempted to determine the effectiveness of continuous intention for users. The study of e-book applications found that readers’ perceptions of playfulness indirectly influenced continuous intentions through satisfaction (Liu et al., 2021). Flow experience has been demonstrated to have a direct influence on ongoing behavior in mobile games, according to research on entertainment and design strategies (Liu et al., 2018). When using first-person view drones as auxiliary devices in matte painting courses, students’ interest in learning is the most important factor that affects continuous learning intentions (Gu et al., 2022c). Thus, this study verifies these three possible influence paths within the interactive library system and proposes design suggestions that can assist in improving continuous learning intentions. Therefore, the following hypotheses are proposed:

*Hypothesis 6 (H6)*: Perceived playfulness in an interactive learning environment can have a significant positive influence on continuous learning intention.

*Hypothesis 7 (H7)*: Flow experience in an interactive learning environment can have a significant positive influence on continuous learning intention.

*Hypothesis 8 (H8)*: Learning interest in an interactive learning environment can have a significant positive influence on continuous learning intention.

## Methodology

### Design and development of systems

**Figure 2:**
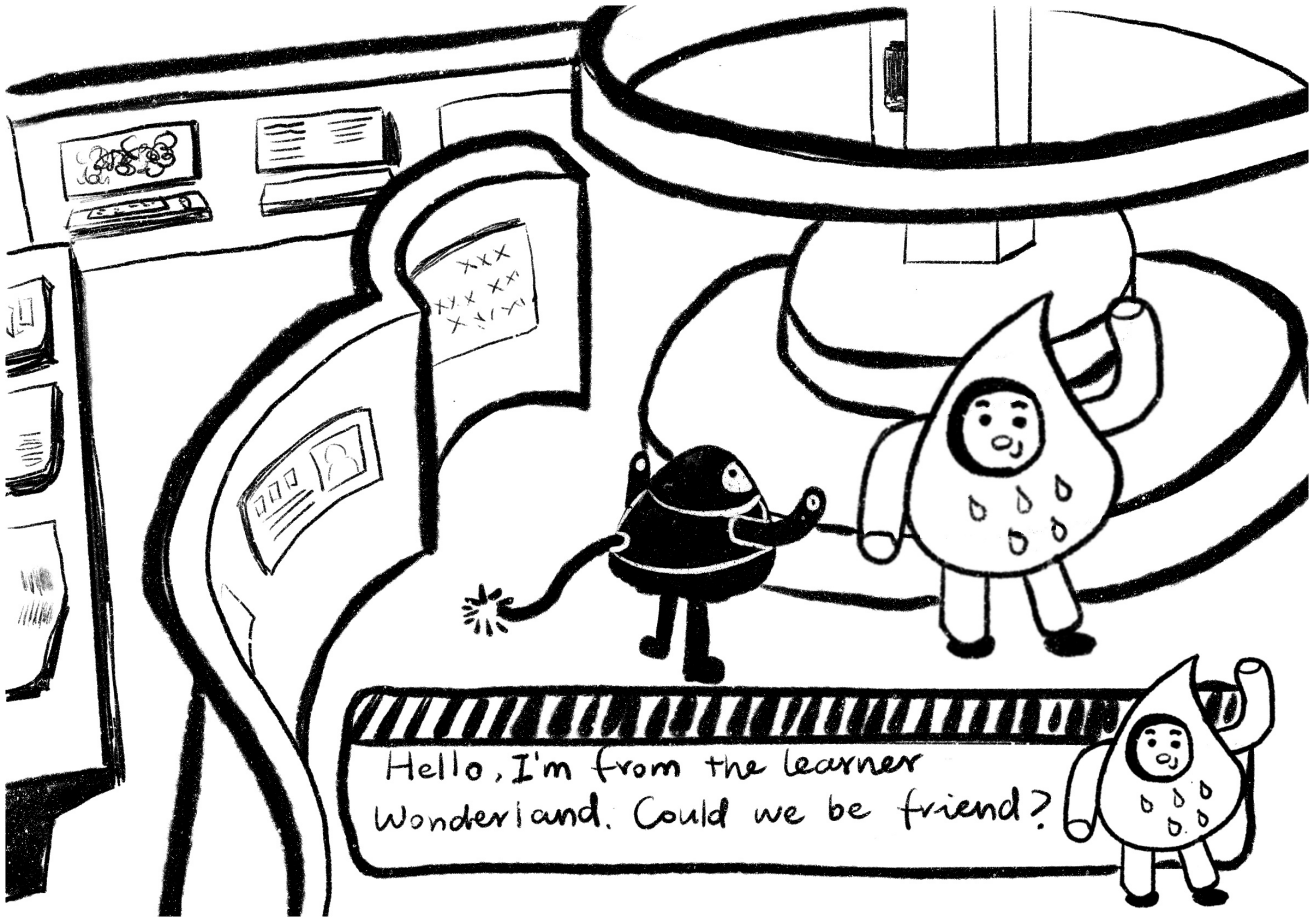
Associating non-player characters (NPCs) with other players.

This study used the role-playing library system developed by Unity as an example of an interactive learning environment. Users are allowed to control the roles that represent themselves within the system. All other characters are non-playable characters. As shown in Figure 2.

**Figure 3:**
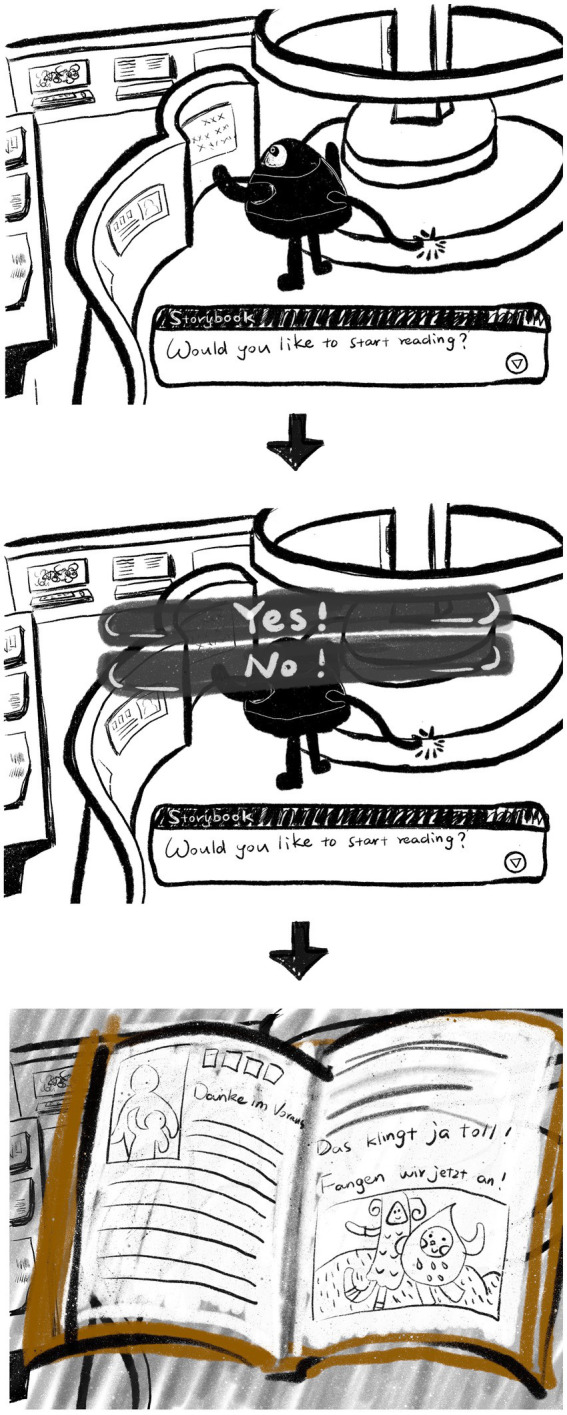
Selection of textbooks to be read.

The reading process is shown in Figure 3. All books in the system are professional German teaching materials, and some books have been enhanced with audio for enhanced learning. We import electronic versions of books in the form of pictures into the library. In the system, the user can open the reading interface by manipulating the character to interact with the book. We set up a transparent quit button on the large blank area to the right of the book. Users can choose to click the book to turn the page or click the quit button to quit reading the current book. The script that provides the exit reading function is shown below.


using System.Collections;
using System.Collections.Generic;
using UnityEngine;
public class PreBtnControl : MonoBehaviour
{
    public void OnClick()
    {
        #if UNITY_STANDALONE // will not do anything while playing in the Unity Editor.
            Application.Quit(); //But it will work in a build.
        #endif
        #if UNITY_EDITOR //  let editor out of play mode
            UnityEditor.EditorApplication.isPlaying = false;
        #endif
    }
}


If the user chooses to continue reading, they can choose to click on the left half of the book and the right half of the book to trigger different page turning directions. Clicking on the left half of the book means turning to the previous page. In particular, if the user is currently reading the first page, there will be no reaction because there is no previous page. The script that triggers the page-turning function by clicking on the left half of the book is shown below.


using System.Collections;
using System.Collections.Generic;
using TMPro;
using UnityEngine;
using UnityEngine.UI;
public class PreBtnControl : MonoBehaviour
{
    // nextBtnControl.cur_page: get the current page
    public NextBtnControl nextBtnControl;
    public Image[] images; // each page of book
    public void OnClick() // will be triggered after click “previous” button
    {
        if (nextBtnControl.cur_page > 0)
        {
            images[nextBtnControl.cur_page].enabled = false; // close now image
            nextBtnControl.cur_page -= 1;
            images[nextBtnControl.cur_page].enabled = true; // show next image
        }
    }
}


If the user chooses to click on the right half of the book, it will turn to the next page. This triggers the emotional support function. If the user reads more than 100 pages, or reads for over 30 min, the system will send a piece of user-care text from the NPC. The text content comes from a large number of statements that we have prepared in advance, selected in a random manner. The selected text will stay in the screen for 10 s and disappear automatically. Audio is played while text is rendered to provide an audio experience. The script that triggers page-turning and emotional support by clicking on the right half of the book is shown below.


using System.Collections;
using System.Collections.Generic;
using TMPro;
using UnityEngine;
using UnityEngine.UI;
public class NextBtnControl : MonoBehaviour
{
    public int cur_page = 0;
    public int pageCnt = 0;
    public float timer = 0f;
    public AudioSource source;
    public List<AudioClip> audioClips;
    public List<string> textList;
    public TMP_Text textVis; // visualization
    private int rand_idx;
    public Image[] images; // each page of book
    public void OnClick() // will be triggered after click “next” button
    {
        if(cur_page < images.Length-1)
        {
            images[cur_page].enabled = false; // close now image
            cur_page +=1;
            images[cur_page].enabled = true; // show next image
            pageCnt += 1; // count the click times of next button.
        }
    }
    IEnumerator showText()
    {
        // show the text corresponding to the audio
        textVis.text = textList[rand_idx];
        yield return new WaitForSeconds(10.0f);
        textVis.text = “”;
    }
    public void Update()
    {
        if (cur_page < images.Length)
        {
            // Turn page 100 times or half an hour passed
            if (pageCnt == 100 || timer >= 1800) // 60*30 sec
            {
                rand_idx = Random.Range(0, textList.Count); // get random index
                source.PlayOneShot(audioClips[rand_idx]); // play the audio
                StartCoroutine(showText()); ; // show text
                // reset
                pageCnt = 0;
                timer = 0f;
            }
            timer += Time.deltaTime;
        }
    }
}


### Research sampling

An online questionnaire was used in this study. We commissioned a market research company to help us recruit participants from various Internet groups across China who were willing to participate in the survey. The questionnaire, which respondents must complete after their experience, includes a link to the learning system. Respondents are required to respond to questions about the educational system in the questionnaire. From April to July 2022, the survey was conducted. In total, 2,377 questionnaires were distributed, and 1,591 valid questionnaires were recovered, with an effective rate of 66.933%. The survey population for this study is a youthful group of people between the ages of 20 and 39 who were recruited by voluntary registration and were subject to age-restrictive requirements. Because there are slightly more female participants who are interested in learning German than male participants, the proportion of female samples recovered in this study is higher than that of male samples. Participants can receive money *via* digital payment after the survey is finished. In Table 1, demographic characteristics of the respondents is presented.

**Table 1:** Demographic characteristics of the respondents.

Sample	Category	Number	Percentage (%)
Gender	Male	689	43.306
	Female	902	56.694
Age	20–29	910	57.197
	30–39	681	42.803
Experience	Yes	1,351	84.915
	No	240	15.085
Monthly Income	Below 4,000	313	19.673
	4,001–8,000	376	23.633
	8,001–16,000	633	39.786
	16,001–30,000	209	13.136
	30,001 or more	60	3.771
Education	Junior high school or below	12	0.754
	High school or secondary school	62	3.897
	Undergraduate or college	1,394	87.618
	Institute including above	123	7.731
Occupation	Civil servant	141	8.862
	Clerk	704	44.249
	Worker	202	12.696
	Public service agency	146	9.177
	Student	229	14.393
	Self-employed	169	10.622
Area	Eastern China	859	53.991
	Central China	392	24.639
	Western China	276	17.348
	Northeast China	61	3.834
	Hong Kong, Macao and Taiwan in China	3	0.189

### Questionnaire design

The questionnaire used in this study was altered in light of prior research. Instead of changing the questioning strategy, this study switched the subject to an interactive learning environment to propose specific questions. To measure emotional support, we adopted five questions from Li (2021). To measure perceived playfulness, this study adopted four questions from Yoo (2021). To measure flow, this study adopted four questions from Lee and Choi (2013). To measure learning interest, this study adopted five questions from Shih and Tsai (2017). To measure continuous learning intention, this study adopted three questions from Chen et al. (2021). The questionnaire used in this study has expert content validity in that it has been reviewed and filled out by three experts in interactive education.

## Results

### Reliability analysis

In this study, SPSS was used to analyze the reliability of the questionnaire. According to the study results, the overall reliability of the questionnaire reached the standard after the deletion of ES3, ES5, PP2, FL2, LI4, and LI5. Hence, the above questions were removed and the remaining data were used for the following analysis and calculation. Table 2 presents the results of the reliability calculation. All items in the remaining data have a corrected item-total correlation (CITC) greater than 0.4. CITC indicators that meet recommended standards indicate the reliability of the project (Zijlmans et al., 2019). While each dimension has a Cronbach’s alpha greater than 0.6, the reliability value obtained after deleting any item is not greater than the current reliability value (Jiang et al., 2022a). To conclude, the results of this study are consistent and suitable for further analysis.

**Table 2:** Description of the test results.

Construct	Coding	Content	Corrected item-total correlation	Cronbach’s alpha if item deleted	Cronbach’s alpha
Emotional support	ES1	NPC in the digital library provide encouragement to me.	0.626	0.754	0.802
	ES2	NPC in the digital library show me empathy.	0.622	0.760	
	ES4	The digital library makes me feel not lonely.	0.701	0.672	
Perceived playfulness	PP1	I feel pleasant when I use the digital library to learn German	0.702	0.728	0.821
	PP3	Using the digital library to learn German is novel and interesting	0.661	0.768	
	PP4	When using the digital library to learn German, I feel happy	0.664	0.765	
Flow	FL1	When using the digital library to learn German, I am not distracted.	0.774	0.831	0.882
	FL3	When using the digital library to learn German, I have a feeling of concentration.	0.783	0.823	
	FL4	When using the digital library to learn German, I do not surf the Internet or things like that.	0.758	0.845	
Learning interest	LI1	The digital library can improve interest in knowledge construction	0.686	0.753	0.824
	LI2	The digital library can improve subject interest	0.674	0.762	
	LI3	The digital library can improve interest in exploring topics	0.682	0.756	
Continuous learning intention	CLI1	I plan to continue to use the digital library to learn German in the future	0.733	0.753	0.841
	CLI2	I plan to use the digital library to learn German often in the future	0.678	0.806	
	CLI3	Generally speaking, I intend to continue to use the digital library to learn German	0.706	0.779	

### Exploratory factor analysis

The validity of the questionnaire was evaluated using exploratory factor analysis. Principal component analysis (PCA) was selected as the method of analysis during the calculation process. Varimax was chose as the rotation method. A summary of the results can be found in Table 3. All dimensions have a Kaiser-Meyer-Olkin (KMO) value greater than 0.5, and the significance of the Bartlett’s Sphere test is less than 0.05, indicating that the data meet the premise of factor analysis (Kaiser, 1974; Norusis, 1998). The further analysis shows that the commonality of each item is greater than 0.5 and that the factor loading is greater than 0.6, which indicates that the dimension has good validity (Osborne, 2008). Furthermore, only one new factor with an eigenvalue greater than 1 can be derived from each dimension, and it has a higher total variation explained. Accordingly, the results of this study meet the standard of single-dimensionality (Kohli et al., 1998).

**Table 3:** Results of reliability analysis.

Construct	Item	KMO	Bartlett’s sphere test	Commonality	Factor loading	Eigenvalue	Total variation explained
ES	ES1	0.699	0.000	0.695	0.834	2.154	71.800%
	ES2			0.687	0.829		
	ES4			0.772	0.879		
PP	PP1	0.717	0.000	0.764	0.874	2.212	73.728%
	PP3			0.722	0.850		
	PP4			0.726	0.852		
FL	FL1	0.745	0.000	0.812	0.901	2.428	80.931%
	FL3			0.820	0.906		
	FL4			0.796	0.892		
LI	LI1	0.722	0.000	0.746	0.864	2.222	74.071%
	LI2			0.734	0.857		
	LI3			0.743	0.862		
CLI	CLI1	0.723	0.000	0.787	0.887	2.278	75.925%
	CLI2			0.731	0.855		
	CLI3			0.760	0.872		

### Confirmatory factor analysis

AMOS was used to conduct confirmatory factor analysis (CFA) in this study. Results indicate that the model achieves the suggested model fit standards (Hair et al., 2006). This study also tested the common method bias of the data by calculating the common latent factor method (CCLFM). According to the results, the model fit results of CCLFM are not significantly superior to those of CFA. CCLFM models reduced RMSEA and SRMR by less than 0.05, while GFI, AGFI, NFI, and CFI models improved by less than 0.1. Thus, the common method bias problem is not present in the data of this study (Lee et al., 2016). Table 4 presents the model fit results of CFA and CCLFM.

**Table 4:** Model fitting index comparison results of confirmatory factor analysis (CFA) and calculating the common latent factor method (CCLFM).

Common indices	*χ*^2^/*df*	RMSEA	GFI	AGFI	NFI	CFI	SRMR
Judgment criteria	< 3	< 0.08	> 0.9	> 0.9	> 0.9	> 0.9	< 0.08
CFA value	2.470	0.030	0.983	0.975	0.984	0.991	0.024
CCLFM value	2.368	0.029	0.984	0.976	0.985	0.991	0.024

The results of the convergent validity test are presented in Table 5. The results indicate that the factor loading of all items is greater than 0.6, and the squared multiple correlation (SMC) is greater than 0.4 (Taylor and Todd, 1995). Furthermore, the average variance extracted (AVE) of each dimension is greater than 0.36, and the composite reliability (CR) is greater than 0.66 (Fernandes et al., 2018). To conclude, all dimensions have good convergent validity.

**Table 5:** Results of the convergent validity test.

Construct	Items	Factor loading	*t* Value	SE	*p* Value	SMC	AVE	CR
ES	ES1	0.742	31.679	0.015	0.002^*^	0.551	0.582	0.806
	ES2	0.706	29.742	0.015	0.001^*^	0.499		
	ES4	0.835	36.818	0.013	0.001^*^	0.697		
PP	PP1	0.823	37.437	0.012	0.001^*^	0.678	0.607	0.822
	PP3	0.762	33.654	0.014	0.001^*^	0.581		
	PP4	0.750	32.892	0.014	0.001^*^	0.562		
FL	FL1	0.844	39.751	0.009	0.001^*^	0.712	0.713	0.882
	FL3	0.877	42.082	0.009	0.001^*^	0.770		
	FL4	0.811	37.556	0.010	0.001^*^	0.658		
LI	LI1	0.790	35.229	0.012	0.001^*^	0.624	0.611	0.825
	LI2	0.777	34.460	0.013	0.001^*^	0.604		
	LI3	0.778	34.524	0.013	0.001^*^	0.606		
CLI	CLI1	0.815	37.243	0.012	0.001^*^	0.665	0.640	0.842
	CLI2	0.776	34.740	0.013	0.001^*^	0.602		
	CLI3	0.809	36.851	0.013	0.001^*^	0.655		

^*^The level of significance is 0.05.

Fornell-Larcker criterion and Heterotrait-monotrait ratio (HTMT) were used to calculate the discriminant validity of the data. As shown in Tables 6 and 7. The results indicate that the square root of the AVE for each dimension is greater than its correlation coefficient with any other dimension. In accordance with the Fornell–Larcker criterion, the recommended standards have been met (Fornell and Larcker, 1981). HTMT results also showed good discrimination between each dimension. All HTMT calculation results are less than 0.8 for each dimension. The study meets the criteria recommended in previous studies (Peralta and Rubalcaba, 2021). Therefore, the dimensions tested in this study have good discriminant validity.

**Table 6:** Results of Fornell-Larcker criterion.

	ES	PP	FL	LI	CLI
ES	0.763				
PP	0.500^*^	0.779			
FL	0.364^*^	0.398^*^	0.844		
LI	0.486^*^	0.593^*^	0.449^*^	0.782	
CLI	0.474^*^	0.603^*^	0.476^*^	0.570^*^	0.800

^*^The level of significance is 0.05.

**Table 7:** Results of Heterotrait-monotrait ratio.

	ES	PP	FL	LI	CLI
ES	/				
PP	0.619	/			
FL	0.432	0.468	/		
LI	0.599	0.720	0.528	/	
CLI	0.577	0.726	0.552	0.685	/

### Modeling structural equations

**Figure 4:**
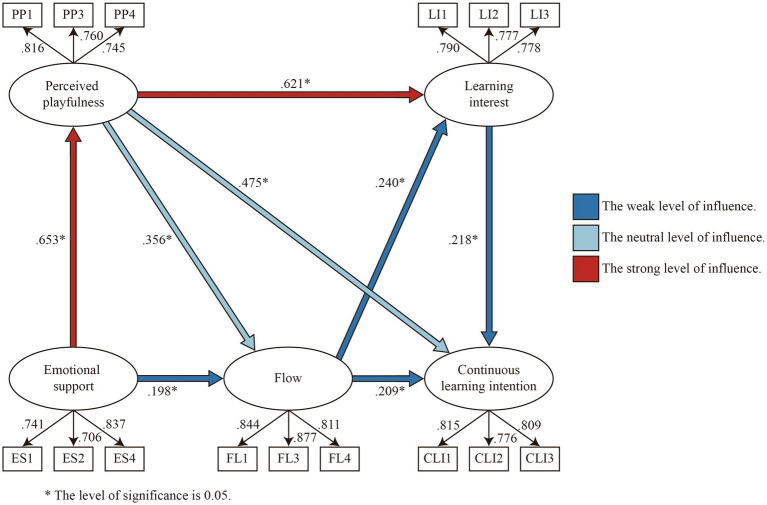
Results of the structural equation model test.

To model the data and evaluate causal relationships, structural equation modeling was used in this study. A total of 2000 Bootstrap runs were conducted with 95% confidence intervals. As shown in Table 8. All fit indices meet the recommended standards, and the results indicate that the model fits well (Hair et al., 2006). Figure 4 illustrates the calculation results for the structural equation model. We have marked the influence degree of the path in the figure. Path coefficient values within the range of 0.1–0.3 represent a weak level of influence, 0.3–0.5 represent the neutral level of influence, 0.5–1.0 indicates the strong level of influence (Owusu et al., 2020). According to the results, each path in the model has reached the significant standard. All of the relational paths established in the model indicate a positive influence relationship between the dimensions.

**Table 8:** Structural equation model fit.

Common indices	*χ*^2^/*df*	RMSEA	GFI	IFI	CFI	TLI	SRMR
Judgment criteria	< 3	< 0.08	> 0.9	> 0.9	> 0.9	> 0.9	< 0.08
Value	2.943	0.035	0.980	0.970	0.981	0.987	0.030

Direct, indirect, and total effect was calculated between the dimensions. As shown in Table 9, the direct influence path coefficient between emotional support and perceived playfulness reaches the significance level and its value is 0.653. Therefore, H1 is supported. In accordance with previous research findings, employees’ perceived playfulness is influenced by their emotional state and how they express that state in work situations (Fang et al., 2019). This study demonstrates the effectiveness of interactive learning by extending an influence relationship to an interactive learning system. In an interactive learning environment, users feel a sense of emotional support, which enhances their enjoyment of learning German.

**Table 9:** Paths affect results.

Hypothesis	Path	Direct effect		Indirect effect		Total effect		Results
		*β*	B-C Sig.	*β*	B-C Sig.	*β*	B-S Sig.	
H1	ES → PP	0.653	0.002^*^	/	/	0.653	0.002^*^	Support
H2	ES → FL	0.198	0.001^*^	0.232	0.001^*^	0.430	0.001^*^	Support
	ES → LI	/	/	0.509	0.002^*^	0.509	0.002^*^	
	ES → CLI	/	/	0.511	0.002^*^	0.511	0.002^*^	
H3	PP → FL	0.356	0.001^*^	/	/	0.356	0.001^*^	Support
H4	PP → LI	0.621	0.001^*^	0.085	0.001^*^	0.707	0.001^*^	Support
H5	FL → LI	0.240	0.001^*^	/	/	0.240	0.001^*^	Support
H6	PP → CLI	0.475	0.001^*^	0.228	0.001^*^	0.704	0.001^*^	Support
H7	FL → CLI	0.209	0.001^*^	0.052	0.001^*^	0.261	0.001^*^	Support
H8	LI → CLI	0.218	0.001^*^	/	/	0.218	0.001^*^	Support

^*^The level of significance is 0.05.

A direct influence path coefficient of emotional support and perceived playfulness on flow experience is 0.198 and 0.356, respectively, both reaching the significant level.

Perceived playfulness as an intermediary variable caused emotional support to have partial mediation on flow, with an influence coefficient of 0.232. Therefore, H2 and H3 are supported.

Previous research found that if a task is enjoyable and fun, it can promote flow experience (Triberti et al., 2021). This point is confirmed in the interactive German library system. Users’ flow states were positively affected by emotional support and perceived playfulness when using the interactive German language learning system.

Learning interest was significantly influenced by perceived playfulness and flow, with path coefficients of 0.621 and 0.240, respectively. The perceived playfulness also produced partial mediation to the learning interest, with an influence coefficient of 0.085. Therefore, H4 and H5 are supported. In accordance with the findings from a previous study, students who learn game design through eye-tracking games report a higher level of flow and therefore increased interest in learning (Gu et al., 2022a). Learning interest is primarily influenced by the user’s perception of the playfulness of the interactive learning system and the flow experience during the learning process. There is a positive correlation between perceived playfulness, flow, and interest in learning and continuous learning intentions. Therefore, H6, H7, and H8 are supported. A direct effect of perceived playfulness is 0.475, and a partial mediation of continuous learning intention is produced under the mediation of flow and learning interest, with an influence coefficient of 0.228. According to previous research on the use of augmented reality, perceived playfulness has a significant influence on flow (Jiang et al., 2022b). In this study, the influence path of the application of interactive learning systems is explored. The influence of interest on the development of a positive and continuous learning state for users is significant. Moreover, flow experience has a direct effect of 0.209 on continuous learning intentions. For continuous learning intention, partial mediation was generated under the mediation of learning interest, with an influence coefficient of 0.052. In previous studies, flow experience has been identified as an important antecedent variable affecting continuous learning intentions when students learn basic modeling design (Hsu et al., 2021). Our research on interactive German library systems demonstrates the importance of helping users to develop a flow experience. Users’ interest in learning in an interactive learning environment is an important factor affecting their commitment to continuous learning. It is consistent with previous research on the factors influencing learners’ desire to continue learning (Gu et al., 2022c). A user’s current learning intention and continuous learning motivation are closely connected.

As shown in Table 10, this study also examined the moderating effects of three moderator variables, including gender, age, and experience. It appears that age plays a significant moderating role in the relationships between more pathways. Gender only moderated the relationship between perceived playfulness and continuous learning intention. Experience only moderated the relationship between perceived playfulness and learning interest.

**Table 10:** Results of mediation effect.

Path	Gender		Age		Experience	
	CMIN	*p*	CMIN	*p*	CMIN	*p*
ES → PP	1.839	0.175	5.994	0.014^*^	1.107	0.293
ES → FL	0.028	0.867	6.195	0.013^*^	0.090	0.765
PP → FL	0.048	0.826	3.298	0.069	0.307	0.580
PP → LI	2.231	0.135	9.366	0.002^*^	5.776	0.016
PP → CLI	4.859	0.028^*^	2.965	0.085	0.522	0.470
FL → LI	0.000	0.987	29.050	0.000^*^	0.047	0.828
FL → CLI	0.032	0.858	2.328	0.127	0.063	0.801
LI → CLI	3.429	0.064	0.009	0.926	3.384	0.066

^*^The level of significance is 0.05.

As shown in Table 11, this study compared the path coefficients that showed significant moderating effects. In the process of generating continuous learning intentions, female users are more sensitive to perceived playfulness than male users. According to our findings, users are more likely to request emotional support as their age increases. It has been observed that older users are more likely to perceive playfulness and flow experiences when they feel emotional support. Learning interest increases as flow experience improves. The path coefficient of young users is higher in the impact path of perceived playfulness on learning interest. According to this study, young users value the system’s noteworthy design. Finally, this study found that subjects with similar usage experience showed a more positive relationship between perceived playfulness and learning interest when experience was used as a moderator variable.

**Table 11:** Comparison between path coefficients with significant moderating effects.

Moderating variable		Path	*β*	*p*
Gender	Male	PP → CLI	0.401	0.001^*^
	Female		0.541	0.001^*^
Age	20–29	ES → PP	0.591	0.001^*^
	30–39		0.729	0.001^*^
	20–29	ES → FL	0.117	0.019^*^
	30–39		0.348	0.001^*^
	20–29	PP → LI	0.695	0.001^*^
	30–39		0.489	0.001^*^
	20–29	FL → LI	0.144	0.001^*^
	30–39		0.416	0.001^*^
Experience	Yes	PP → LI	0.637	0.001^*^
	No		0.529	0.001^*^

^*^The level of significance is 0.05.

## Discussion

The purpose of this study is to investigate users’ perceptions of emotional support, perceived playfulness, flow, learning interest, and continuous learning intention in an interactive German learning environment by developing a role-playing library system. This study estimated the relationship between dimensions using structural equation modeling and tested the effects of gender, age, and experience as moderators.

According to our research, emotional support is crucial for users learning German in the library system (corresponding to supported H1 and H2). In addition, an NPC can serve as a source of emotional support. NPC’s emotional support is positively correlated with user’s perceived playfulness, flow, and learning interest as well as indirect effect on continuous learning intentions in students. The findings of this study reduce the social costs that students must bear in order to obtain emotional support. Previous research revealed that online collaborative tools enhance communication and emotional support between students and are beneficial for educational purposes (Hernández-Sellés et al., 2019). There are also some studies that show that teachers’ emotional support contributes significantly to students’ positive learning status and learning effectiveness (Kikas and Tang, 2019; Malloy, 2019). Therefore, educators and designers of educational tools often focus on ways to foster a sense of caring among their students. There is, however, a strong correlation between emotional support from real people and students’ perceived social anxiety and social competence. Due to their own growth environment, students behave differently in a social environment (Ren and Li, 2020). It is important for researchers to pay close attention to the social anxiety problems that students may experience while learning online (Keskin et al., 2020). It is, therefore, necessary to fully understand and stimulate students’ social skills as well as to carefully design courses in order to accomplish the emotional support provided by interpersonal interaction. This study proposes and demonstrates that emotional support from NPCs enhances learning at low levels of socialization. This discovery offers a new way for the creation of a human-computer interactive learning environment. Furthermore, the ability to converse and exchange with avatars may even be beneficial for reducing social anxiety (Rasouli et al., 2022). Based on this information, it may be concluded that the use of virtual characters’ emotional support as a means of assisting students’ learning may actually contribute to the development of their social skills, such as their ability to communicate and interact with others. By utilizing the NPC system, the traditional method of teaching-learning interaction can be improved. When comparing users between the ages of 20–29 and 30–39, we also found that emotional support in an interactive learning environment was more helpful in learning German for older users. It may be that as users age, they become more dependent on companionship and psychological comfort due to the pressures of work and family responsibilities. Mental health can be maintained and improved with emotional support (Green et al., 2021). Therefore, this study believes that in order for interactive German learning to be effective, the designers of the system should pay close attention to the human-computer interaction provided by NPC. It is particularly important to consider the perceptions and preferences of relatively older users when designing the user experience. The necessity of receiving emotional support in learning is further shown by the fact that caring from NPCs might somewhat lessen students’ unfavorable view when they do not receive emotional support from teachers or peers. Educators should pay more attention to students’ psychological pressure and emotional status, give students more understanding and encourage students to communicate with each other. Teachers can also try to introduce more cutting-edge programs to act as NPCS in interactive teaching and provide emotional support. The above findings likewise suggest that while investigating human-computer interaction in education, researchers should attempt to reorient their attention away from widely debated notions and toward strategies for inciting students’ pleasant feelings. It is beneficial to better assist the digital revolution of education if there is in-depth theoretical building of potential issues with inadequate emotional support in education.

As demonstrated in the system, perceived playfulness has a strong positive influence on learning status (corresponding to supported H3, H4 and H6). Besides affecting flow directly, perceived playfulness can also influence learning interest and continuous learning intentions indirectly. During the COVID-19 pandemic, online education offers flexibility in terms of time and space. However, some teachers believe that online education is not suitable for foreign language instruction, as it causes students to lose motivation and seriousness during the learning process (Celik et al., 2022). There is some debate concerning whether interactive teaching systems should emphasize the importance of seriousness in classroom learning as a means of achieving teaching objectives. This study provides a solution to this problem. In user research on mobile learning technologies, it has been shown that perceived playfulness is closely related to students’ intention to continue learning (Huang et al., 2021). By adding more interesting elements to the design of the education system, users are more likely to engage in long-term learning. In this study, the results support this research conclusion and extend it to an interactive learning environment for German language learning. This study also found that perceived playfulness has a positive effect on learning especially among female users, younger users, and users who have experience using similar interactive systems. It is important for interaction designers to consider the preferences of the corresponding user groups and launch certain features to enhance the fun for these users. In addition to the design strategy, according to the research results, teachers can try more interesting teaching actively, such as adding puzzles, time-limited answering games, points competition games and other common fun teaching methods in the classroom. The combination of these methods and interactive education can help teaching activities. Interactive education environment can meet the equipment needs for fun. Teachers should learn more about digital education technology in order to take full advantage of the more possibilities that interactive technology brings to education.

Lastly, this study demonstrates the positive effects of flow experiences on students’ learning outcomes (corresponding to supported H5, H7 and H8). There has been some concern expressed in previous studies that students may ignore the learning content as they become too engrossed in the state of flow and lose track of time and space (Triberti et al., 2021). Interaction between human and computers can contribute to the perception of interactivity, vividness, and novelty in learning environments (Gu et al., 2022c). It appears that these feelings are more related to technology than learning content. Based on the above findings, this view cannot be accepted. This study found that the flow experience successfully improved students’ learning interest and continuous learning intention. Through the application of more cutting-edge interactive technologies, interaction designers can further enhance the flow experience of the teaching system. Our research suggests that interactive technology can be beneficial for education, echoing some of the research that supports this claim. For example, augmented reality teaching significantly increases students’ motivation to learn and their flow experience (Wang et al., 2022). The flow experience has a positive effect on students’ willingness to continue learning through MOOCs (Zhao et al., 2020). Due to the positive feelings they instill in students, interactive learning and flow experiences are not detrimental to teaching goals.

## Conclusion

### Theoretical contribution

**Figure 5:**
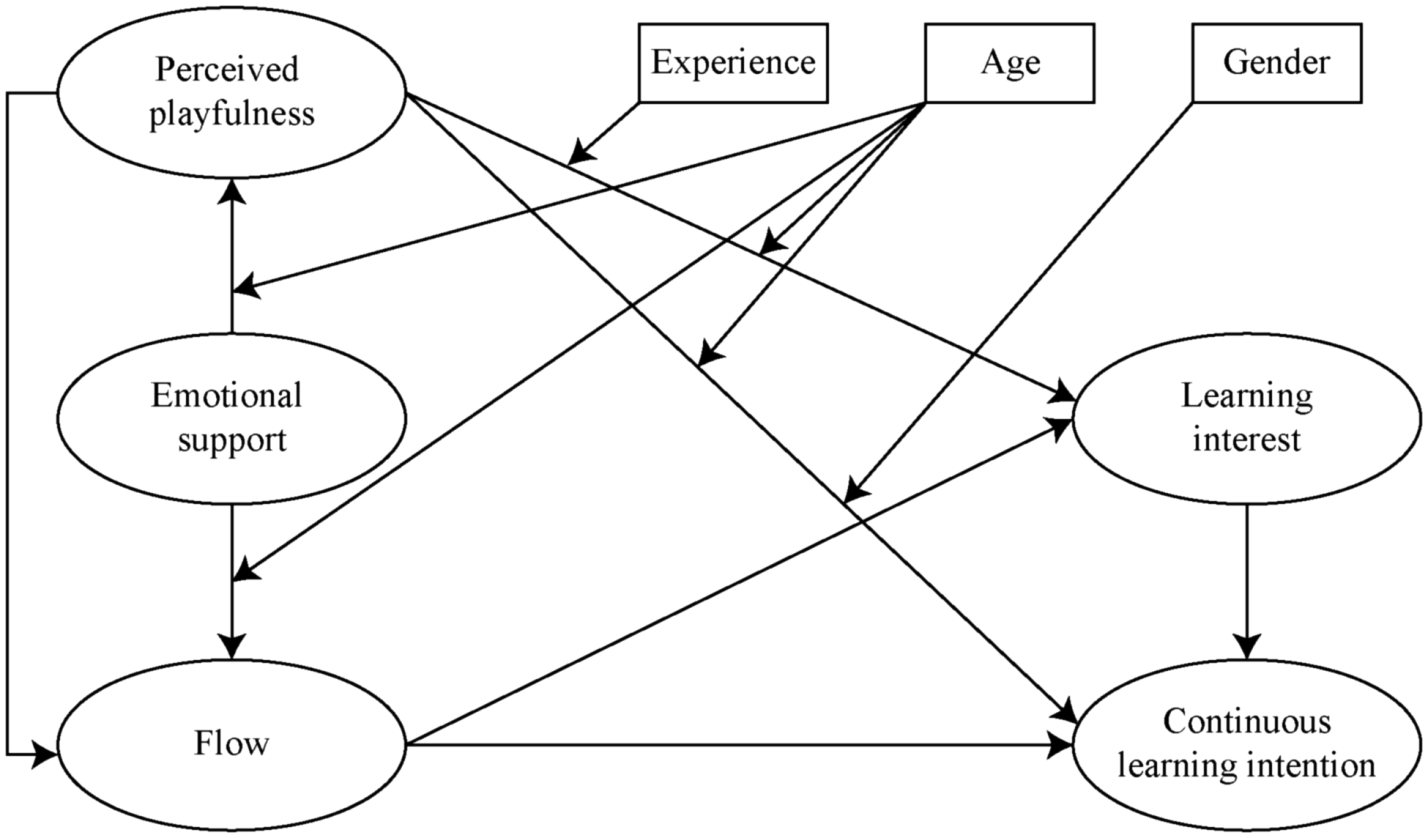
Emotion-driven learning model (ELM).

This study demonstrates that emotional support and perceived playfulness play an important role in determining the users’ learning status in an interactive German learning system that utilizes NPCs to provide empathy and care. Through interactive learning, the user’s flow experience, learning interest, and continuous learning intention are all enhanced. According to this study, the influence paths of emotional support on perceived playfulness and flow were antecedents to other dimension relationships. Based on these findings, this study developed a quantitative model that we named emotion-driven learning model (ELM), as shown in Figure 5. Just as perceived ease of use as a source in the technology acceptance model has an impact on each dimension of the theory (Hua and Wang, 2019). The model is named for the evidence that emotional support contributes to interactive education, and that emotional support positively affects other dimensions within our hypothetical framework, either directly or indirectly.

As with some other theories of interactive education, this model focuses on improving students’ flow experience and learning status. For instance, the technology incentive model (TIM) and the interactive narrative design for learning model (INDLM; Gu et al., 2022b,c). The results of our study confirm that emotional support and perceived playfulness are important aspects that cannot be overlooked in the study of interactive education system design. Additionally, as in the extended unified theory of acceptance and use of technology (UTAUT2), this study found that gender, age, and experience have significant moderating effects on user behavior (Tamilmani et al., 2021). Particularly, age as a moderator showed a broader moderating effect, inferring that younger and older groups perceived the interactive system differently. It has been found that the path coefficient of perceived playfulness is higher in the test results of young groups. In the test results of the elderly population, emotional support significantly impacted the dependent variable. In only one influence pathway, gender and experience had significant moderating effects, both of which involved perceived playfulness. The results of this study indicate that even though perceived playfulness can have a positive and direct impact on learning status for all users, female users who have had interactive learning experiences are more likely to think that perceived playfulness has a significant influence on learning status.

### Practical implications

Based on the findings of this study, recommendations are provided for the design of educational systems. This study illustrates two important points. As a first point, emotional support provided by NPCs to users can have a very positive impact on German learning, just as caring and empathy from real people can have a positive effect. In the subsequent development of interactive learning systems, the findings of this study provide theoretical support for the feasibility of incorporating virtual learning partners. In addition, perceived playfulness should be added to the system in a way that does not negatively impact learning interest and continuous learning intentions by interfering with the serious classroom atmosphere. It is important for interaction designers to enhance the system’s perceived playfulness and emotional support. A further finding of this study is that users weigh different factors differently, even though their overall preferences are consistent. Users can be divided into two distinct categories according to the degree to which their preferences differ from one another. The first group consists of older users, who demonstrate a need for the system to provide them with emotional support. The second group consists of young women with experience using interactive learning systems. The group places a great deal of emphasis on perceived playfulness of the system. A strong interest in playfulness was expressed by them. Accordingly, this study recommend that two strategies be developed in the system design and in actual teaching based on the target audience or the number of students in the class. The first strategy involves learning ways to gain emotional support at the same time. This can be accomplished by including the teacher’s deliberate attention, encouraging empathy between the teacher and students, and adding the emotional support function of NPCs to the interactive system. These methods can be utilized to create an atmosphere of positive emotions in the classroom, such as warmth, listening, encouragement, and empathy. Second, the strategy aims to improve the perceived playfulness of learning. The course design can be made interesting by adding interesting content, elements, and interaction in the system. Through these two different strategies of teaching or system design, students are able to achieve a better learning experience and better learning outcomes.

## Limitations and future research

In this study, three research limitations are identified that could be investigated and analyzed further in the future to further explore and supplement theoretical gaps:

1. *The limitations of respondents*. All 2,377 subjects who participated in our survey were Chinese. The age range for these individuals is between 20 and 39 years of age. Future research can be conducted to verify the effectiveness of the ELM model proposed in this paper in other different countries and regions. As we only surveyed users aged 20–39, the differences in the model reflected by age are limited to the segmentation results between the younger groups. In the future, younger and older users may be investigated to determine whether their perceptions and preferences are compatible with the theoretical framework developed in this study.
2. *The limitations of learning content*. This study conducted a survey only among users of German language learning. Verified results can only reflect the user’s perception when learning German. This theoretical model may be applicable to a wider range of interactive educational situations. So, in the future, the study of other foreign languages will be able to be carried out in conjunction with the study of other subjects that are not limited to language education.
3. *The limitations of learning tools*. This study conducted an in-depth investigation on the perceived playfulness and emotional support from NPCs in our study, and adopted the role-playing library system developed by ourselves in order to effectively reduce the interference from other factors. To verify the accuracy of the theoretical framework of this research, future research can be applied to other systems used in education today, such as MOOCs, virtual reality technology, and augmented reality technology.

## Data availability statement

The raw data supporting the conclusions of this article will be made available by the authors, without undue reservation.

## Ethics statement

The studies involving human participants were reviewed and approved by the review committee of the Ministry of Social Science, Changshu Institute of Technology. The patients/participants provided their written informed consent to participate in this study.

## Author contributions

JS and CG: conceptualization. YY, TC, SL, and JC: data curation. SL and HS: formal analysis. WM: investigation. TC and WW: methodology. YY and JC: project administration. JL and WW: software. HS: validation. CG: writing-original draft. JL, WM, and JS: writing-review and editing. All authors contributed to the article and approved the submitted version.

## Funding

This work was supported by the Special Research Project on World Languages and Cultures for Young and Middle-aged Teachers’ Education and Research in Fujian Province (grant number JSZW22002).

## Conflict of interest

The authors declare that the research was conducted in the absence of any commercial or financial relationships that could be construed as a potential conflict of interest.

## Publisher’s note

All claims expressed in this article are solely those of the authors and do not necessarily represent those of their affiliated organizations, or those of the publisher, the editors and the reviewers. Any product that may be evaluated in this article, or claim that may be made by its manufacturer, is not guaranteed or endorsed by the publisher.
